# Confounding effect of socioeconomic position in the study of height in relation to prostate cancer risk

**DOI:** 10.1038/sj.bjc.6601799

**Published:** 2004-04-06

**Authors:** G D Batty

**Affiliations:** 1 Department of Social Medicine, Panum Institute, University of Copenhagen, Blegdamsvej 3, DK-2200 Copenhagen N, Denmark

Sir,

In their report, Engeland *et al* (2003) examined height (an indicator of genetic and early life environmental factors) and body mass index (an indicator of overweight) in relation to prostate cancer, finding an elevated risk in men of tall stature and of overweight. While the study offers high power (a total of 33 314 verified cases in a cohort of almost one million men), there was an absence of any collateral data.

Both height and overweight are socially patterned, with the highest proportion of taller and leaner adult individuals among the more affluent (Batty and Leon, 2002). Socioeconomic position is also associated with prostate cancer mortality, generally with an elevated risk in the higher social groups (Davey Smith *et al*, 1991; Pukkala and Weiderpass, 2002). This raises the question of whether socioeconomic differences in height and overweight may be an alternative explanation for their apparent relation with prostate cancer risk. While recent evidence suggests this is not the case for overweight in relation to prostate cancer (Calle *et al*, 2003), two studies (Leon *et al*, 1995; Nilsen and Vatten, 1999) that explored the predictive value of height for this malignancy found that the magnitude of the association was attenuated following control for social factors which included educational attainment and occupational social class. All observational studies are hampered by some methodological shortcoming; however, confounding by socioeconomic position is important in the study of cancer aetiology, and may explain the apparent protective effect of short stature on prostate cancer in the present study.
